# Patients, clinicians and open notes: information blocking as a case of epistemic injustice

**DOI:** 10.1136/medethics-2021-107275

**Published:** 2021-05-14

**Authors:** Charlotte Blease, Liz Salmi, Hanife Rexhepi, Maria Hägglund, Catherine M DesRoches

**Affiliations:** 1 General Medicine and Primary Care, Beth Israel Deaconess Medical Center, Boston, Massachusetts, USA; 2 School of Informatics, University of Skövde, Skovde, Västra Götaland, Sweden; 3 Department of Women's and Children's Studies, Uppsala Universitet, Uppsala, Sweden; 4 Harvard Medical School, Boston, Massachusetts, USA

**Keywords:** patient perspective, autonomy, public health ethics, clinical ethics

## Abstract

In many countries, including patients are legally entitled to request copies of their clinical notes. However, this process remains time-consuming and burdensome, and it remains unclear how much of the medical record must be made available. Online access to notes offers a way to overcome these challenges and in around 10 countries worldwide, via secure web-based portals, many patients are now able to read at least some of the narrative reports written by clinicians (‘open notes’). However, even in countries that have implemented the practice many clinicians have resisted the idea remaining doubtful of the value of opening notes, and anticipating patients will be confused or anxious by what they read. Against this scepticism, a growing body of qualitative and quantitative research reveals that patients derive multiple benefits from reading their notes. We address the contrasting perceptions of this practice innovation, and claim that the divergent views of patients and clinicians can be explained as a case of epistemic injustice. Using a range of evidence, we argue that patients are vulnerable to (oftentimes, non-intentional) epistemic injustice. Nonetheless, we conclude that the marginalisation of patients’ access to their health information exemplifies a form of epistemic exclusion, one with practical and ethical consequences including for patient safety.

## Introduction

Today, via secure web-based portals, many patients can rapidly and conveniently access their medical records including the very words written by clinicians (hereafter, ‘open notes’). In some countries, such as Estonia, the Nordic countries and the USA, open notes are advanced.^1^ For example, in Sweden, most patients are already offered open notes via ‘Journalen’, one of the eHealth services on the nationwide patient portal. In the USA, from 5 April 2021 (postponed from 2 November 2020 due to COVID-19) new federal rules mandate that, on request and with few exemptions, all health organisations offer open notes to patients.^2 3^ Worldwide, however, there is still limited uptake of the practice. For example, in Canada and Germany, open notes are available to some patients but not yet offered universally. In the UK in April 2020, it was announced that patients in National Health Service (NHS) England will be offered access to general practitioners’ clinical notes, although on a prospective basis.^4^

Against these developments, a growing body of research reveals a range of ethical and potential health and safety benefits of patient access to their clinical notes, including greater engagement and boosting recall about their care plans,^5–10^ which we discuss in this paper. However, research also points to notable hesitancy, and even averseness, to the practice among some health professionals.^11 12^ Moreover, in comparison with other services offering consumers and the public access to their secure personal information, such as online banking, the implementation of open notes in healthcare remains comparatively slow.

We argue that the resistance of clinicians and health organisations to sharing clinical notes, amounts to an ethical concern that can best be understood using Miranda Fricker’s concept of epistemic injustice.^13^ According to Fricker, the sharing and production of knowledge is a valued good, and as such, epistemology is interlinked with ethical considerations. Inequalities in the access, production or participation in knowledge formation can constitute an ethical wrong, leading to primary or secondary harms. Fricker terms these harms as ‘epistemic injustice’. Applying this concept to healthcare, scholars have argued that this framework provides a useful approach for understanding distinctive epistemic injustices in the formation of, for example, medical curricula, the dynamics of health encounters and in health policy-making.^14–18^

In this paper, we apply this theoretical framework to open notes. We reflect on the ethical and epistemic repercussions of denying patients rapid and convenient online access to their clinical notes, and on the differences between patient and clinician perspectives on this practice innovation. Drawing on a range of surveys, we propose that there is evidence to substantiate the claim that clinicians negatively stereotype patients in ways that unfairly deflate their credibility. Furthermore, negative characterisations of patients are often offered by clinicians as a justification withholding access to notes. However, as we argue, evidence indicates that patients may suffer disadvantages or possible harms, due to lack of access to their clinical information.

At the outset, we emphasise that there are numerous reasons why health organisations in different countries and providers, might deny patients access to open notes. Our concern in this paper is specifically with evidence of epistemic injustice as it pertains to examples of health professionals’ resistance, reluctance or misgivings about offering the majority of adult patients open notes.

We begin with a brief overview of open notes, including how the practice differs from patients’ requests to obtain copies of their clinical records. Following this, we outline Fricker’s account of epistemic injustice which describes how spheres of social activity are entwined with epistemic and ethical dimensions. In this section, we define testimonial injustice and hermeneutic injustice which can be interpreted, respectively, as giving information/knowledge to others, and making sense of one’s experiences.^16^ As Fricker, and as other philosophers and health researchers have argued, these injustices may undermine healthcare professionalism, leading to risks of patient harm.^15 19–21^

Next, using Fricker’s framework as our foundation, we present evidence from quantitative and qualitative studies of clinicians’ and patients’ experiences and attitudes about open notes. We draw on findings from across medical specialties, including primary care, oncology and psychiatry. In addition, and where appropriate, we document events, some of which are matters of public record. Using this evidence, we propose that, under the explicit or implicitly voiced assumption that ill persons cannot handle access to their clinical notes, patients are subject to forms of epistemic subordination which may incur health disadvantages. Furthermore, structural disadvantages with respect to patients’ access to their notes may diminish the epistemic resources available to clinicians which, in turn, may result in patient harms. The paper concludes with recommendations on how clinicians, health organisations and eHealth designers might strive to reduce epistemic injustice in this domain.

## Background on open notes

In many countries, patients already have the legal right to request access to their clinical records. In the USA, for example, The Health Insurance Portability and Accountability Act of 1996 gave patients the right to obtain copies of their clinical notes. Under this legislation, providers could levy a fee for the administrative costs of access, and requests were usually expected to be fulfilled within a ‘reasonable time frame’ of 30 days.^22^ Similarly, in the UK in 2000, the Freedom of Information Act allowed patients to request access to information held by public authorities such as the NHS. Open notes are different. Neither a software nor a product, the innovation is similar to accessing one’s personal financial information via online banking. Open notes facilitate rapid, convenient access to clinical notes via digital devices such as smart phones, laptops and tablets.

Currently, patients in around 10 countries worldwide are offered access to some, or all, of their clinical notes.^1^ In this paper, we focus on survey findings from Sweden and the USA, where multiple recent studies into the practice have been conducted. In Sweden, access to clinical notes was launched in 2012, when the region of Uppsala gave all citizens over 18 years of age online access to their notes. In this first region-wide trial, the patients were offered access to their notes through an online patient portal called ‘Journalen’.^23 24^ Then, in 2015, Journalen was launched as the national system in Sweden for online access to clinical notes. At the end of 2018, all regions had implemented Journalen and today, in a population of around 10 million, more than 4.5 million citizens have accessed their clinical notes.

Meanwhile, in the USA, from April 5 2021, new federal law mandated that all providers give patients immediate electronic access to their clinical notes on request. It permits ‘information blocking’ of medical notes if doing so ‘…will substantially reduce the risk of harm’ to a patient or to another person (§171.201(a) p. 704).^2^ Licensed health professionals can decide what constitutes a substantial risk when working ‘…in the context of a current or prior clinician–patient relationship’ (p. 702). Under the new ruling, the burden will be on providers to justify why they have blocked information from patient view.

It is important to emphasise that in certain circumstances caution around open notes may sometimes be justified. We fully agree that, in specific contexts, it may be ethically justified to withhold patient access to clinical notes.^25–27^ For example, it would be justifiable to temporarily block access if patients in domestic abuse situations inform their clinician that they are at greater risk of harm from a controlling partner reading their clinical notes. Many ethical questions also arise with respect to proxy access, including provisions for adolescents,^28^ and older people who are vulnerable such as those living with advanced stage dementia. As with other innovations in healthcare, open notes may bring benefits while also inviting new ethical dilemmas, and these emergent practice challenges will need to be resolved. Notwithstanding, our focus is the decision to offer or deny the wider adult patient population access to their clinical notes.

In the remainder of this paper, we suggest that implicit and explicit stereotyping of patients contributes to concerns, reluctance, or even unwillingness, among many to share notes with patients (‘testimonial injustice’). This injustice, in turn, may lead to reduced access to healthcare resources which might otherwise aid patients in understanding and managing their illness or condition (‘hermeneutic injustice’). To develop these claims, we must first examine Fricker’s account of these concepts in greater detail.

## Epistemic injustice

Epistemic injustice points to a specific kind of injustice done to someone in their capacity as a knower, or as a contributor to knowledge. According to Fricker, this injustice takes two forms, testimonial and hermeneutical, which we discuss below. Although debate has arisen among epistemologists about other kinds of epistemic injustice,^19 29^ our focus in this paper is on Fricker’s framework which remains widely accepted among philosophers of medicine.^15 20^

### Testimonial injustice

According to Fricker, testimonial injustice arises when an individual, such as a speaker, is unfairly attributed a lower level of credibility because of discrimination—typically, because of their membership of a negatively stereotyped group. In such circumstances, the conversation partner or listener, implicitly and/or explicitly interprets the speaker to have diminished capacity as both a bearer and contributor to knowledge. For example, the speaker may be considered unreliable or untrustworthy. As a result of implicit or explicit negative stereotyping, he or she may be unjustly excluded from shared epistemic activities, or their contributions may be ignored or downgraded to a lower status. Importantly, not all forms of stereotyping are unjustified. In medical practice, for example, clinicians rely on heuristics and stereotypes for certain presentations of illnesses. Such reliable generalisations are crucial for physicians to make diagnoses. Rather, Fricker refers to cases of discriminatory, negative stereotyping with respect to some aspect of an individual’s identity (eg, gender, age, accent, race/ethnicity, disability or personality). The listener, Fricker notes, withholds proper respect for the speaker who suffers, whether knowingly or not, a degradation that also constitutes an ‘epistemic insult.’

In healthcare, a growing body of literature proposes that patients may be vulnerable to testimonial injustice.^15 20 21 30^ Deflation of patients’ credibility can constrain their meaningful contribution to clinical visits, and wider dialogue with the healthcare community about their health condition. As Kidd and Carel note, ‘pre-emptive derogation of the epistemic credibility and capacities of ill persons’ amounts to the ‘a priori view, for instance, of ill persons being confused, incapable or incompetent, that distorts an evaluation of their actual epistemic performance’.^19^ In this way, patients may even be viewed as susceptible to psychological fragility or irrationality, and their testimonies may be dismissed as, irrelevant, emotional, unhelpful or time-consuming.^15 20^ Worth noting, even in contexts where clinicians are genuinely sympathetic, they may still fail to perceive ill persons’ contributions as worthy of epistemic consideration.^15^ For example, an elderly patient may be treated with compassion but routinely overlooked, or infantilised in visits in favour of discussing the patient’s health with an attendant family or friend caregiver.^31 32^ A wealth of research shows that some patient populations are at greater risk of negative stereotyping, and as a result, may be subjected to epistemic subordination in visits.^32–34^

We emphasise that epistemic injustice does not challenge the legitimate epistemic authority that some individuals, such as medical doctors, have acquired as a result of extensive and highly specialised training.^16^ Rather, the concept of epistemic injustice invites a wider discussion about the kinds of epistemic privilege that exist and the contributions that may be constrained or delimited across a range of epistemic activities in healthcare.^15^ For example, patients have first-person experiences of living with an illness or disability, which means that they may have acquired deep insights about their symptoms, medical treatments and the nature of healthcare delivery.^19 35^ Some persons with illness may also develop considerable medical knowledge after intensively researching their condition, and the contributions of patients to medicine can be valuable in shaping critical conversations and debate.^36–39^ Equally, clinicians may accrue deeper appreciation and insights about healthcare delivery by experiencing life as a patient.^40–42^ In summation, as Blease *et al* argue, ‘Injustice arises with respect to epistemic privilege when one group fails to recognise the unique expertise of another group, or when an individual fails to fully appreciate the epistemic contributions of another individual’.^16^

On the other hand, an important consideration is unwarranted epistemic privilege. In healthcare contexts, this may arise when clinicians, researchers or patients are credited with having a level of knowledge or insight beyond their knowledge and/or experience. This can happen, for example, if medical doctors are assumed by patients to have specialised expertise about a disease beyond their formal education or ongoing training. Nor are all ill persons epistemically reliable. Some illnesses may interfere with cognitive judgements and personal insights. Yet even in these scenarios, patients may still be vulnerable to epistemic injustice since negative stereotyping on the part of clinicians may interfere with judgements about the patient’s capacity as a knower, and as a contributor to knowledge-formation activities.^15 16^

### Hermeneutical injustice

Deficits in access to shared epistemic resources represent another form of injustice. Fricker defines this as ‘hermeneutic injustice’ which she describes as a structural and social problem that occurs when, ‘both speaker and hearer are labouring under the same inadequate tools’.^13^ It arises when individuals cannot access, or can only partially access, resources that could better support interpretation of their experiences. Failure to access these conceptual resources may asymmetrically affect one group of individuals, conferring a structural disadvantage on them. Impoverished access may also arise because one group restricts collective access to other groups, or when one group has ‘perfectly adequate hermeneutic resources of its own’ but contributions are ignored or not deemed credible by a dominant group.^20^

As Fricker notes, and as other researchers have proposed, hermeneutic injustice can also arise in healthcare where, ‘social and healthcare cultures have features that impede the hermeneutic agency of ill persons’.^19^ Current hermeneutical structures and practices can play a role in enhancing (or diminishing) patients’ experiences and understanding of their illness. Obtaining a diagnosis, for example, can help patients to transform a confusing or puzzling set of symptoms into an understandable illness, sometimes with an aetiological explanation, and opportunities to avail of possible therapeutics. Despite familiar rhetoric in modern healthcare about the importance of eliciting the patient perspective in clinical practice and research, structures and existing practices may sustain hermeneutical injustice by delimiting access to resources which might help patients to understand, or directly contribute to research into, their illness or condition.

Kidd and Carel describe two kinds of ‘strategies’ that may underpin hermeneutical injustice both of which may be consciously or non-consciously endorsed.^20^ ‘Strategies of exclusion’ refer to ‘excluding a currently hermeneutically marginalised group from the practices and places where social meanings are made and legitimated, such as professional committees or legislative bodies’.^20^ This can take a variety of forms, including physical exclusion to, ‘subtler forms of epistemic exclusion, such as the procedural insistence on the employment of strenuous legal, medical or academic terminologies and conventions so as to exclude those who are not members of those groups from participating in deliberative processes.’^20^

A related concern is ‘strategies of expression’ which Kidd and Carel describe as the demotion of modes of expression that are not routinely adopted by the dominant group. Such modes of expression may include anecdotal, autobiographical or affective styles of presentation, which may be interpreted as evidence of the lack of rationality or diminished capacity of the marginalised group. According to Kidd and Carel, in their efforts to be heard the oppressed group may strive for further recognition. However, their efforts may be interpreted as ‘pushy’ or as further evidence of ‘irrationality’ which, in turn, may prompt greater frustration driving further ‘epistemic disenfranchisement’.^16 20^ As a net result of these two strategies of exclusion ill persons may suffer, ‘a double injury’.^15 20^ Patients may be excluded from sense-making activities in healthcare on the basis of unfair prejudice leading to a vicious cycle: in this way, it is argued, ‘hermeneutical injustice (exclusion from the structural processes of knowledge formation) may also intensify testimonial injustice and vice versa.’^16^

## Using epistemic injustice to understand attitudes to open notes

### Evidence from clinicians

In Sweden and the USA, survey research shows that many clinicians, including those with no prior experience of open notes, are often deeply sceptical or resist the idea of open notes, believing that most patients will be confused or anxious if they read their notes which will directly lead to a rise in patient contact and greater workplace burdens.^11 43–46^ Such negative stereotyping of patients as perplexed, emotional or as ‘time-wasters’, appears to form a common rationale for resisting open notes or delaying implementation of the practice. Shortly, we will provide evidence that these assumptions constitute a form of testimonial injustice. First, it is important to note the strength of clinicians’ negative feelings about the disruptive potential of patient access.

In Sweden, the introduction of Journalen led to strong and mostly negative reactions from healthcare professionals, especially from physicians and their trade union.^24^ During the implementation of Journalen in the Uppsala region, where the practice was first pioneered in Sweden, physicians not only expressed their concerns to the implementation team, they went to great length to stop the project which was reported to nine different authorities, including the Swedish Work Environment Authority, in attempts to hinder patients from gaining online access to their notes.^47^

Negative stereotyping was also evidenced recently in the USA. Following the announcement about the new federal rules mandating patient access to their clinical notes, in many publicly accessible social media posts in forums on medical topics, clinicians voiced strong resistance to the prospects of patient access. For example:

There are zero positives to this from our perspective…I definitely think this is one of the cases where the pendulum has swung way too far in the direction of autonomy rather than paternalism after overcorrecting for the crappiness of the past.^48^ [Reddit contributor identifying as a medical resident. Post ‘liked’ 435 times].

*‘*We’ve been doing [open notes] for a while and I see zero effect…[E]ven those patients/families that do read the notes still have no clue what is going on. It’s like showing someone a bunch of stock market numbers and thinking they can predict the ebb and flow of the economy.’^49^ [Reddit contributor identifying as an ‘RN’ [registered nurse] in the ICU. Post liked 3 times].

An article describing the new rules on the US website ‘Medscape’ which covers news and resources for physicians prompted 138 comments.^50^ Some comments were highly sceptical about the capacity of patients to understand their notes, and to derive value from them; for example:

The average patient reads at the 4th to 6th grade level. Are we to write two levels of notes, to accommodate patients who may not have even finished 8th grade? Another reason for me to formalize my retirement plan.

In other high-income countries, patients have access to some of their record. For example, in the UK patients who access their care through NHS England can use health portals to read lists of medications, test results and appointments and referral dates. For most British citizens, the majority of the notes written by specialist physicians remain hidden from view. In 2020, writing in the BMJ, and wary of the prospects of patient access one physician remarked: *‘*Im [also] concerned about what might not be written in the notes in future, the things we’ll leave out for fear of upset. Observations about the mismatch between symptoms and signs, concerns about self-care, possible but unlikely diagnoses that I should check up on—these all feature in my GP notes but would need a whole load of explaining to patients.’^51^

A considerable body of survey research challenges the negative stereotype of confused or emotionally fragile patients requiring more contact time with clinicians—a form of labelling, we suggest that constitutes an ‘epistemic insult’. For example, using objective measures of messaging—such as email volume—in 2012 a US survey by Delbanco *et al* found that patient contact did not change significantly in the 12 months before compared with the 12 months after open notes was implemented.^43^ After experience with open notes, workload concerns among participating physicians also diminished markedly. Study authors reported that some physicians were so surprised by the lack of disruption that, ‘Several wondered whether the intervention had been implemented’. A more recent US survey found that, among clinicians who had opened their notes for at least 1 year, 86% (n=1112) reported that in the previous 12 months, patients contacted them less than monthly or never with questions about their notes.^52^ Similar findings have emerged from mental healthcare where after implementing open notes, mental health clinicians report minimal workflow disruptions.^7 44^ Countries or health organisations that deny, delay or impede access, on the basis that patients might be ‘time-wasters’ or unreliable or untrustworthy readers of their notes, exemplify testimonial injustice.

However, there is also evidence that testimonial injustice engenders hermeneutic injustice. The latter arises when patients are prevented from deriving benefits from their notes and potentially correcting errors in them. Studies show that after accessing their open notes, patients can, and do, perceive mistakes in their documentation.^53 54^ For example, in the USA, in the large-scale survey of more than 22 000 patients who read their notes, around one in five reported finding an error with 40% perceiving the mistake as serious.^53^ The most common errors included mistakes in diagnoses, medical history, medications and test results. Notably, in one study, of the patients who reported contacting their physician about an error in their notes, 85% (231/331) reported satisfaction with how the matter was resolved.^55^ Yet by excluding patients from readily accessing and offering feedback on their notes, both patients and clinicians may be, in Fricker’s language, ‘labouring under the same inadequate tools’.^13^ When health organisations and clinicians deny patients access to their clinical information, clinicians are thereby deprived of a key resource (namely, patients) who might help improve on their documentation.

Supporting this view, qualitative findings from psychiatry and psychotherapy surveys also show that some patients identify inaccuracies in the reporting about their subjective or emotional states.^7 56^ As one psychotherapy patient noted, *‘*Interpretations of feelings are just that, someone else’s attempt to understand. They are not always correct. The note is a permanent and sometimes incorrect portrayal of a discussion.’^56^ Although we know of no large-scale surveys aimed at investigating whether patients perceive errors in mental health notes, soliciting patients’ insights may be valuable for correcting or improving precision in clinician’s judgements about patients’ first-person states.^26^ Lacking access to their notes and the possibility of picking up on errors, omissions, or inaccuracies, these findings strongly point to a form of, what Fricker terms, ‘cognitive disablement’—patients are structurally prevented from obtaining convenient, rapid access to their notes—preventing them from partnering with clinicians to improv the accuracy of their records.

Even when open notes are implemented, how clinicians use portal features may impose strategies that continue to exclude patient contributions. For example, in Sweden, there is no obligation on clinicians to read patients’ comments on notes submitted via Journalen. In this way, patients’ contributions remain subject to what Kidd and Carel describe as ‘an epistemically marginal role in consultative exercises’.^20^ Additional evidence of strategies of exclusion are found in a recent US study^52^ which reported that, after opening notes to patients, the majority of physicians (78%, n=620) admitted that they did not encourage patients to read their notes.

Language used in clinical documentation may impose subtler strategies that may still exclude the full epistemic engagement of patients. In a study conducted with mental health clinicians at the VA, responding to whether they had made or will make changes to the way they document mental health notes as a result of patient access, 29% (n=45) of surveyed clinicians reported they would write less about the diagnosis.^44^ Similarly, existing forms of medical expression in notes may stifle patient collaboration.^52^ In a recent web-based survey in the USA, 58% (n=422) of physicians reported changing, ‘use of language that could be perceived as critical of the patient’ with 41% (n=306) reporting that they changed the ‘use of terms such as ‘noncompliant,’ ‘patient refuses’ and ‘patient denies.’’ While this points to mindfulness in record-keeping among some clinicians, among the majority the continued use of medical vernacular appears to signal traditional roles of ‘authoritative doctors’ and ‘submissive’ or ‘disobedient patients’. In a recent survey, Fernández *et al* found that 11% (n=2411) patients who accessed their notes felt judged or offended by what they read, which included forms of labelling and disrespectful language.^57^ Choice of words, therefore, may provide further unintentional discrediting of patients, which may serve as a barrier to their epistemic engagement, which in turn may engender patient distrust, possibly leading to patients’ withdrawl from care.^58^ The net result is that the quality of epistemic resources may become further impoverished, reducing the potential of notes to aid patients in understanding and managing their condition (thereby, illustrating hermeneutic injustice).

### Evidence from patients

The evidence of both testimonial and hermeneutic injustice when it comes to clinical information blocking is strengthened by findings from patient surveys. In particular, the starkly contrastive evidence of patients’ experiences with open notes compared with clinicians’ perceptions of patients’ experiences provide compelling evidence of unjustified negative stereotyping about the capacity of individuals to understand or emotionally cope with what they read. Despite physicians’ doubts about patients’ competency and their emotional rectitude to read their notes, in recent quantitative and qualitative surveys of patients’ experiences with open notes, the overwhelming majority of respondents reported positive experiences.^5 8 43^ In surveys from Sweden and the USA, around 98% of surveyed patients with experience of the practice believed open notes are a good idea or reported they wanted access to continue.^5 7 8 43^ For example, in a large scale survey research of over 22 000 patients in the USA, Walker *et al* found that only a small minority—3% and 5%—of patients reported being very confused or more anxious by what they read.^5^ Also worth noting, it is undetermined whether reported patient anxiety reached diagnostic thresholds. Conceivably, for example, respondents’ anxiety might have been the result of being better informed about their health condition and not a result of open notes per se. However, most patients may not recognise they are the victims of testimonial injustice when it comes to the failures of health organisations in their country to offer them ready access to their online notes. This is in part because many patients may not be aware of the innovation, or know of the ongoing debates about patient access including that many clinicians resist the idea.

Data provide evidence that testimonial injustice gives rise to informational deficits that constitute hermeneutic injustice. In a range of surveys, patients report multiple, hitherto unexperienced, benefits from accessing their notes. For example, in the survey by Walker *et al*, of patient respondents who read at least one visit note in the last 12 months, the majority of surveyed patients described feeling more in control of their healthcare, enhanced understanding of the rationale for treatments and referrals, better remembering their treatment plans, and, as a result, doing a better job taking their medications.^5 59^ We suggest that inherent informational gaps in clinical record access are tantamount to forms of hermeneutic injustice because patients are routinely excluded from making greater sense of their clinical diagnoses, and from better understanding and engaging in their own treatment plan.

Next, we review evidence of epistemic injustices pertaining to particular patient populations.

#### Marginalised patient populations

Studies show that minorities, persons with low incomes, older patients and those who do not speak the same language as their provider, are more vulnerable to implicit negative biases on the part of providers which may contribute to increased likelihood of communication breakdowns among these patient populations.^33^ Relatedly, such patients may suffer a ‘double injury’ when it comes to information blocking. Perhaps because they are vulnerable to nonconscious forms of epistemic discrediting, and communication breakdowns, such patients may accrue greater benefits from accessing their notes away from the pressures and limitations of the face-to-face encounter.^60^ Supporting this interpretation, survey research from the USA, shows that, compared with their counterparts, patients who are older, in poorer health, persons with fewer years of formal education, minorities, and those whose first language is not English, are significantly more likely to report that open notes boost their recall, understanding, and engagement in their care plan.^5 6 61^ However, research in the USA indicates that the likelihood of receiving an access code to activate a health portal is lower among already disadvantaged patient groups.^62^ Such persons may be relegated lower credibility as knowers and as a result experience impoverished access to their clinical information (‘testimonial injustice’). Furthermore, without adequate safety nets in health systems targeted at improving access among these populations, patients may routinely miss out on particularly important benefits of reading their notes (‘hermeneutic injustice’).

#### Patients with mental health conditions

The degree of clinician scepticism about patients’ capacity to understand and cope with reading their notes is particularly salient among patients with mental health conditions. For example, in a Swedish study, over half of surveyed mental health clinicians (58%, n=488) anticipated that, ‘a majority of mental health patients will worry more’.^11^ Around one in two (53%, n=438) believed that, ‘a majority of patients will find the notes more confusing than helpful’ with fewer than a third (30%, n=252) expecting that, ‘a majority of patients will better understand their health and medical conditions’ as a result of accessing their notes.^11^ Similarly negative attitudes have emerged in US surveys. For example, a survey of mental health clinicians at the Department of Veterans’ Affairs (VA)—the nationwide healthcare system that offers veterans portal access to their clinical notes—67% (n=135) believed that, ‘a majority of patients will find the notes more confusing than helpful’, while 77% (n=156) of participants anticipated that ‘a majority of mental health patients will worry more’.^44^ A growing body of research suggests that clinicians’ views amount to what Kidd and Carel describe as ‘pre-emptive derogation of the epistemic credibility and capacities of ill persons’. In a recent US study of open notes in primary care, there was no reported difference between the experiences of patients with and without a mental health diagnosis, with most patients reporting that they felt more in control of their healthcare (92%, n=336 of patients with a mental health diagnosis compared with 91%, n=1789 without). Only a small minority—1% (n=5) of patients with a mental health diagnosis compared with 3% (n=49) of those without—reported finding the notes more confusing than helpful.^63^ In a pilot study of 52 patients at an outpatient psychiatric clinic in Boston, USA, only a few patients reported being confused or anxious by what they read.^7^

Drawing on these findings, it seems reasonable to postulate that clinicians’ a priori stereotyping of all mental health patients as incapable of handling their notes is unjustified and constitutes a form of testimonial injustice. The injustice arises because a range of policy decisions and covert practices have been implemented to selectively withhold clinical information from this patient population. For example, in the USA, psychotherapy notes are exempt from the new rules about sharing.^2 3^ In Sweden, only around half of the country’s 21 regions share notes from psychiatric clinics. In Norway, all patients are offered access to open notes but a survey of psychiatry clinicians working in hospitals found that 8% maintained a ‘shadow record’ precluding some patients from reading some details about their health.^64^

Again, there is also apt evidence that such testimonial injustice gives occasion to hermeneutic injustice. The injustice arises because patients are denied valuable opportunities to better understand, remember and engage with information about their treatment plan. In Canada, in 2020, a qualitative study reported that psychiatry patients who accessed their information were better able to identify patterns related to their mental health which, in turn, provided greater sense of control over their illness.^65^ Failure to take medications and stick to treatment plans is huge challenge in healthcare, and one that especially effects patients with mental health diagnoses. A study in 2020 found that around 50% of patients with include major depressive or bipolar disorders, or schizophrenia failed to take their prescription medications.^66^ A recent survey analysis found that persons with serious mental illnesses—defined as including major depression, bipolar disorders and schizophrenia disorders—were significantly more likely than other patients to report feeling in control of their medications, to understand adverse effects, and to report taking their medications, after accessing their notes.^67^

Even while acknowledging that there may be occasions when some patients with mental illnesses may be upset, or confused by what they read, or that some patients may even be harmed by access,^25 26^ this growing body of research indicates that the majority of mental health patients derive benefits from reading their clinical notes. When health organisations deny mental health patients access to resources—namely, their clinical notes—this constitutes a form of hermeneutic injustice since it may impede patients’ capacity to understand their clinical condition and treatment plan, and potentially to improve their health outcome.^68^

#### Patients accessing cancer care

In cancer care, many clinicians believe that online access to oncology notes and test results pose special difficulties for patients. The deflation of cancer patients’ epistemic credibility is particularly well illustrated by a recent US study. Analysing survey results, Salmi *et al* found that 98% (n=3366) of patients with a cancer diagnosis who read their visit notes agreed open notes was a ‘good idea’ compared with 70% (n=70) of oncology clinicians who had opened their notes.^69^ More starkly, only 4% (n=131) of oncology patients reported finding their notes confusing, compared with 36% (n=44) of oncology clinicians who believed their patients would find the notes ‘more confusing than helpful’ and 27% (n=33) who did not know if patients found notes confusing. In a recent research study conducted in Sweden with oncology healthcare professionals 6 years after the launch of Journalen, Moll and Cajander reported that the majority of both physicians and nurses believed that clinical notes were more confusing than helpful for patients.^70^ Qualitative research provides further evidence of the epistemic derogation of cancer patients among clinicians with the claim that blocking access is justified. In a study by Grünloh *et al* oncologists expressed concerns about patient’s ability to understand test results, advising that withholding notes and test results was warranted.^46^ For example:

I have a long education, and I do tests and other things and then I put all these things together…I do not want anyone to put their nose in this… I think it can be very dangerous if the patient comes in during this investigation and sees the test results;

They are not able to interpret the test results, and it leads to more work, revisits and telephone calls, and they are worried. /…/ A thing like this is nothing but a lot of extra work /…/. I see nothing positive in that the patients read their medical records online;

Some can become calmer actually, when they have seen the test results, and know what they are. But not the oncologic patients, they are mostly worried.

We suggest that oncology clinicians’ opinions that patients are ill equipped to read their notes, and that blocking or delaying opportunities to access results is therefore justified, amounts to a case of testimonial injustice. While Swedish users of Journalen prefer immediate access, and rate test results as one of the most important information types to which they can have access,^8^ strategies of exclusion are apparent. Under the assumption that patients might be too emotional, or lack the necessary rational capacity to choose whether or when to access their test results, many patients are still subjected to a form of epistemic insubordination. Some regions, for example, impose a 2-week embargo and others only release results once they have been checked by a physician.^71^

When oncology patients lack timely access to their clinical resources (namely, to the patients’ own oncology test results and notes) this can undermine understanding about their diagnosis and treatment plan (‘hermeneutic injustice’). For example, in Uppsala University Hospital, Uppsala, Sweden, where Journalen has been implemented since 2012, many oncology patients reported feeling more in control of their care than when they were not offered access to their notes, with many citing rapid access to test results as essential for safeguarding their mental well-being.^9^ In the study by Rexhepi *et al* patients attested, *‘*Accessing test results, it is a tremendous difference, and it really means a lot to me…It’s so difficult to wait, whether it is good news or bad news, it’s very good to know’; ‘I think that the information that you have been diagnosed with cancer is worrying no matter how you get it… I think that we should be free to choose how we get access to that information’. Or as one patient bluntly put it: ‘if we can manage to have all of these cancer diseases and to live with it, then we can handle reading about it’.

Patients living with cancer who are not offered the choice to access to their test results and oncology notes may experience cognitive disablement. Under pressurised, and oftentimes upsetting face-to-face visits, patients may be unable to retain or process all the information that their clinician is communicating.^72^ Studies of patient portal access in Sweden and the USA demonstrate that by ‘extending the visit’ beyond traditional, one-shot disclosures during appointments, patients with cancer better remember and understand crucial information about their diagnosis and prognosis.^9^ For example, in a US survey in radiotherapy, 60% (53/88) of oncology patients accessed their notes when given the option, and all patients who chose to read their notes found them to be useful.^73^ Among those radiotherapy patients who accessed their notes, 96% (51/53) reported better understanding their diagnosis, 94% (50/53) reported better understanding of the treatment risks and side effects, and 91% (48/53) described learning important information they had missed during visits. These survey findings are illustrative of hermeneutic injustice since, with limited or no access to their notes, many patients with cancer may be denied opportunities to better understand and manage their care.

### Summary

The conclusion we draw, based on the contrasting findings of research into open notes among clinician and patients, is that patients—including those with serious illnesses—may be vulnerable to unjustified negative stereotyping with respect to their capacity to understand and cope with reading their clinical notes. We emphasise that some patients in only around ten countries worldwide are currently offered access to their notes.^1^ Anticipating that patients may be too emotional or that access will lead to work burdens, clinicians and health organisations appear to have resisted open notes or express serious misgivings about shared access.^51^ Drawing on a growing body of survey research, we find robust evidence that strategies designed to exclude or limit access to their notes can give rise to hermeneutic injustice. When patients are unable to, or not encouraged to readily access their notes they lose out on important opportunities to support understanding and interpretation of their diagnosis and care plan.^5 7 55 59 67^ In countries where open notes are not yet implemented, policies that preserve the ‘epistemic isolation’ of patients, forfeit chances for individuals (or their family members) to benefit from, and collaborate with clinicians to actively contribute, and improve, clinical documentation.^20^

Merely offering open notes will not be sufficient to address all the existing hermeneutic shortfalls. For example, it seems likely that many patients remain unaware that they can access their clinical notes.^74^ In the USA, a recent survey found the majority of clinicians did not encourage patients to read their notes.^52^ In Sweden, there is still no obligation on clinicians to read patient feedback submitted via portals. Here again, by relegating patients’ contributions to a lower epistemic status or ignoring their input, clinicians thereby miss out on key chances to improve the quality of their record-keeping and augment their epistemic performance, potentially improving patient care.^54^

## Conclusions and recommendations

Pioneering physician Warner Slack once opined that patients are the ‘largest and least used resource in healthcare’.^75^ Research into open notes supports the wisdom of this insight. When patients are systematically denied or offered only restricted access to their notes, this can also compromise the epistemic activities of both patients and clinicians. Patients’ active engagement with their documentation presents an important, and hitherto underappreciated mechanism to strengthen patient-clinician teamwork, improve diagnostic processes and prevent errors.^54^

Our first recommendation is that health organisations strive to provide the electronic infrastructure to support open notes for patients. However, merely offering open notes is unlikely to reap all the potential benefits of patients as active epistemic partners. Second, therefore, we recommend that clinicians explicitly encourage patients to read their notes, and offer feedback on possible errors, omissions and inaccuracies^74 76^ For example, in the USA, a web-based educational programme for veterans offered by the VA providing guidance on reading mental health notes proved highly effective in engaging patients and promoting trust in clinicians.^77^ Portal innovations which actively solicit feedback, such as the implementation of ‘Our Notes’^78^—which allows patients to cocreate their clinical notes—may serve to close the feedback loop on care, and function as a patient safety mechanism.^79 80^ Such strategies could develop a richer, patient-focused agenda using the words and forms of expression of the patient.

Third, since not all patients are able to access electronically housed clinical notes, beyond providing access to WiFi and portal-enabled electronic devices, we recommend health organisations invest in new outreach programmes aimed at training patients in basic digital literacy skills, such as how to use the internet, logon to portals, and read their own health data.^81^

Fourth, there is an important role for eHealth designers and informaticians working in collaboration with health organisations and—crucially—patients, to develop structural improvements in patient portals. Such designs should encompass notes in multiple languages, and interfaces that allow manually impaired and sight-impaired patients to access and contribute to their clinical notes.^82^ The use of tooltips or other design modifications embedded in clinical notes may also facilitate patient understanding of medical or psychotherapy terminology without further compromising the clinician’s time and workload.^60 83^

Finally, clinical education can play a role in improving practitioners’ education about open notes, and the justifications for sharing notes with patients. Indeed, it is our hope that this paper will prompt a deeper discussion about the role of epistemic injustice in healthcare, and a call to action. Specifically, we hope that health professionals will reflect on the ethical justifications of sharing notes, the epistemic and healthcare advantages of engaged patient partners and the harms of withholding patient access.

## References

[1] Essén A , Scandurra I , Gerrits R , et al . Patient access to electronic health records: differences across ten countries. Health Policy Technol 2018;7(1):44–56. 10.1016/j.hlpt.2017.11.003

[2] Health and Human Services Department, USA . 21St century cures act: Interoperability, information blocking and the onc health it certification program, 2020. Available: https://www.federalregister.gov/documents/2020/05/01/2020-07419/21st-century-cures-act-interoperability-information-blocking-and-the-onc-health-it-certification [Accessed 15 Jul 2020].

[3] Blease C , Walker J , DesRoches CM , et al . New U.S. law mandates access to clinical notes: implications for patients and clinicians. Ann Intern Med 2021;174(1):101–2. 10.7326/M20-5370 33045176

[4] Richards T . Light amid the gloom. The BMJ opinion, 2020. Available: https://blogs.bmj.com/bmj/2020/03/12/tessa-richards-light-amid-the-gloom/ [Accessed 8 Apr 2020].

[5] Walker J , Leveille S , Bell S , et al . OpenNotes after 7 years: patient experiences with ongoing access to their clinicians' outpatient visit notes. J Med Internet Res 2019;21(5):e13876. 10.2196/13876 31066717 PMC6526690

[6] Bell SK , Folcarelli P , Fossa A , et al . Tackling ambulatory safety risks through patient engagement: what 10,000 patients and families say about Safety-Related knowledge, behaviors, and attitudes after reading visit notes. J Patient Saf 2018. 10.1097/PTS.0000000000000494. [Epub ahead of print: 27 Apr 2018]. 29781979

[7] Peck P , Torous J , Shanahan M , et al . Patient access to electronic psychiatric records: a pilot study. Health Policy Technol 2017;6(3):309–15. 10.1016/j.hlpt.2017.06.003

[8] Moll J , Rexhepi H , Cajander Åsa , et al . Patients' experiences of accessing their electronic health records: national patient survey in Sweden. J Med Internet Res 2018;20(11):e278. 10.2196/jmir.9492 30389647 PMC6238103

[9] Rexhepi H , Åhlfeldt R-M , Cajander Åsa , et al . Cancer patients' attitudes and experiences of online access to their electronic medical records: a qualitative study. Health Informatics J 2018;24(2):115–24. 10.1177/1460458216658778 27440056

[10] Kipping S , Stuckey MI , Hernandez A , et al . A web-based patient portal for mental health care: benefits evaluation. J Med Internet Res 2016;18(11):e294. 10.2196/jmir.6483 27852556 PMC5131190

[11] Petersson L , Erlingsdóttir G . Open notes in Swedish psychiatric care (Part 1): survey among psychiatric care professionals. JMIR Ment Health 2018;5(1):e11. 10.2196/mental.9140 29396386 PMC5816262

[12] Petersson L , Erlingsdóttir G . Open notes in Swedish psychiatric care (Part 2): survey among psychiatric care professionals. JMIR Ment Health 2018;5(2):e10521. 10.2196/10521 29929946 PMC6035347

[13] Fricker M . Epistemic injustice: power and the ethics of knowing. Oxford University Press, 2007.

[14] Carel H , Györffy G . Seen but not heard: children and epistemic injustice. The Lancet 2014;384(9950):1256–7. 10.1016/S0140-6736(14)61759-1 25289422

[15] Carel H , Kidd IJ . Epistemic injustice in healthcare: a philosophial analysis. Med Health Care Philos 2014;17(4):529–40. 10.1007/s11019-014-9560-2 24740808

[16] Blease C , Carel H , Geraghty K . Epistemic injustice in healthcare encounters: evidence from chronic fatigue syndrome. J Med Ethics 2017;43(8):549–57. 10.1136/medethics-2016-103691 27920164

[17] Crichton P , Carel H , Kidd IJ . Epistemic injustice in psychiatry. BJPsych Bull 2017;41(2):65–70. 10.1192/pb.bp.115.050682 28400962 PMC5376720

[18] Younas A . Epistemic injustice in health care professionals and male breast cancer patients encounters. Ethics Behav 2020;61:1–11. 10.1080/10508422.2020.1756819

[19] Kidd IJ , Carel H . Healthcare practice, Epistemic injustice, and Naturalism. Royal Institute of Philosophy Supplement 2018;84:211–33. 10.1017/S1358246118000620 32997467

[20] Kidd IJ , Carel H . Epistemic injustice and illness. J Appl Philos 2017;34(2):172–90. 10.1111/japp.12172 28303075 PMC5324700

[21] Kidd IJ , Medina J , Pohlhaus Jr G . The Routledge Handbook of epistemic injustice. Taylor & Francis, 2017.

[22] US Department of Health and Human Services . Health insurance portability and accountability act of 1996, 1996. Available: https://www.hhs.gov/sites/default/files/privacysummary.pdf [Accessed 26 Aug 2019].

[23] Rexhepi H , Moll J , Huvila I . Online electronic healthcare records: comparing the views of cancer patients and others. Health Informatics J 2020;26(4):2915–29. 10.1177/1460458220944727 32914701

[24] Scandurra I , Jansson A , Forsberg-Fransson M-L . Patient accessible EHR is controversial: lack of knowledge and diverse perceptions among professions. International Journal of Reliable and Quality E-Healthcare 2017;6:29–45.

[25] Strudwick G , Yeung A , Gratzer D . Easy access, difficult consequences? providing psychiatric patients with access to their health records electronically. Front Psychiatry 2019;10:917. 10.3389/fpsyt.2019.00917 31920763 PMC6926439

[26] Blease CR , O'Neill S , Walker J , et al . Sharing notes with mental health patients: balancing risks with respect. Lancet Psychiatry 2020;7(11):924–5. 10.1016/S2215-0366(20)30032-8 32059796

[27] Mehta S , Jamieson T , Ackery AD . Helping clinicians and patients navigate electronic patient portals: ethical and legal principles. CMAJ 2019;191(40):E1100–4. 10.1503/cmaj.190413 31591096 PMC6779538

[28] Bourgeois FC , DesRoches CM , Bell SK . Ethical challenges raised by OpenNotes for pediatric and adolescent patients. Pediatrics 2018;141(6):e20172745. 10.1542/peds.2017-2745 29776979

[29] Hookway C . Some varieties of epistemic injustice: reflections on Fricker. Episteme 2010;7(2):151–63. 10.3366/epi.2010.0005

[30] Kidd IJ . Epistemic injustice, healthcare, and illness: a bibliography. Epistemic injustice, healthcare, and illness: a bibliography, 2020. Available: https://ianjameskidd.weebly.com/epistemic-injustice-healthcare-and-illness-a-bibliography.html [Accessed 12 Dec 2020].

[31] Adelman RD , Greene MG , Charon R . The physician-elderly patient-companion triad in the medical encounter: the development of a conceptual framework and research agenda. Gerontologist 1987;27(6):729–34. 10.1093/geront/27.6.729 3428622

[32] Adelman RD , Greene MG , Ory MG . Communication between older patients and their physicians. Clin Geriatr Med 2000;16(1):1–24. 10.1016/s0749-0690(05)70004-5 10723614

[33] FitzGerald C , Hurst S . Implicit bias in healthcare professionals: a systematic review. BMC Med Ethics 2017;18(1):19. 10.1186/s12910-017-0179-8 28249596 PMC5333436

[34] Arpey NC , Gaglioti AH , Rosenbaum ME . How socioeconomic status affects patient perceptions of health care: a qualitative study. J Prim Care Community Health 2017;8(3):169–75. 10.1177/2150131917697439 28606031 PMC5932696

[35] Carel H . Illness: the Cry of the flesh. Routledge, 2018.

[36] Riggare S . Patient researchers - the missing link? Nat Med 2020;26(10):1507 10.1038/s41591-020-1080-4 33029015

[37] Salmi L , Brudnicki S , Isono M , et al . Six countries, six individuals: resourceful patients navigating medical records in Australia, Canada, Chile, Japan, Sweden and the USA. BMJ Open 2020;10(9):e037016. 10.1136/bmjopen-2020-037016 PMC7493106 32933961

[38] Kindlon T . Reporting of harms associated with graded exercise therapy and cognitive behavioural therapy in myalgic Encephalomyelitis/Chronic fatigue syndrome. Bull IACFS ME 2011;19:59–111.

[39] Kindlon T . Do graded activity therapies cause harm in chronic fatigue syndrome? J Health Psychol 2017;22(9):1146–54. 10.1177/1359105317697323 28805516

[40] Rosenbaum EE . A taste of my own medicine: when the doctor is the patient. Random House New York, 1988.

[41] Kalanithi P . When breath becomes air. Random House, 2016.

[42] Awdish R . In shock: how nearly dying made me a better intensive care doctor. Random House, 2018.

[43] Delbanco T , Walker J , Bell SK , et al . Inviting patients to read their doctors' notes: a quasi-experimental study and a look ahead. Ann Intern Med 2012;157(7):461–70. 10.7326/0003-4819-157-7-201210020-00002 23027317 PMC3908866

[44] Dobscha SK , Denneson LM , Jacobson LE , et al . Va mental health clinician experiences and attitudes toward OpenNotes. Gen Hosp Psychiatry 2016;38:89–93. 10.1016/j.genhosppsych.2015.08.001 26380876

[45] Grünloh C , Myreteg G , Cajander Åsa , et al . "Why Do They Need to Check Me?" Patient Participation Through eHealth and the Doctor-Patient Relationship: Qualitative Study. J Med Internet Res 2018;20(1):e11. 10.2196/jmir.8444 29335237 PMC5789160

[46] Grünloh C , Cajander Åsa , Myreteg G . "The Record is Our Work Tool!"-Physicians' Framing of a Patient Portal in Sweden. J Med Internet Res 2016;18(6):e167. 10.2196/jmir.5705 27349531 PMC4940602

[47] Lyttkens L . Sustains: support users to access information and services, report D6.1 v1.1, 2014. Available: https://projectdome.files.wordpress.com/2021/01/sustains-rapport.pdf [Accessed 20 Jan 2021].

[48] MD - PGY-6 Pulm Research Fellow . Thoughts on open notes legislation. Reddit, 2020. Available: https://www.reddit.com/r/medicine/comments/ituxxb/thoughts_on_open_notes_legislation/g5gulom/?utm_source=reddit&utm_medium=web2x&context=3 [Accessed 20 Jan 2021].

[49] RN - ICU . Thoughts on open notes legislation? Reddit, 2020. Available: https://www.reddit.com/r/nursing/comments/iz7s5u/thoughts_on_open_notes_legislation/g6iu3x8/?utm_source=reddit&utm_medium=web2x&context=3 [Accessed 20 Jan 2020].

[50] Mulcahy N . Patients can read your clinical notes starting nov 2. Medscape, 2020. Available: https://www.medscape.com/viewarticle/939499 [Accessed 20 Jan 2021].

[51] Salisbury H . Helen Salisbury: whose record is it anyway? BMJ 2020;368:m753. 10.1136/bmj.m753 32127408

[52] DesRoches CM , Leveille S , Bell SK , et al . The views and experiences of clinicians sharing medical record notes with patients. JAMA Netw Open 2020;3:e201753 10.1001/jamanetworkopen.2020.1753 32219406 PMC12124735

[53] Bell SK , Delbanco T , Elmore JG , et al . Frequency and types of patient-reported errors in electronic health record ambulatory care notes. JAMA Netw Open 2020;3(6):e205867 10.1001/jamanetworkopen.2020.5867 32515797 PMC7284300

[54] Blease CR , Bell SK . Patients as diagnostic collaborators: sharing visit notes to promote accuracy and safety. Diagnosis 2019;6(3). 10.1515/dx-2018-0106 31039128

[55] Bell SK , Mejilla R , Anselmo M , et al . When doctors share visit notes with patients: a study of patient and doctor perceptions of documentation errors, safety opportunities and the patient-doctor relationship. BMJ Qual Saf 2017;26(4):262–70. 10.1136/bmjqs-2015-004697 PMC7255406 27193032

[56] O'Neill S , Chimowitz H , Leveille S , et al . Embracing the new age of transparency: mental health patients reading their psychotherapy notes online. J Ment Health 2019;28(5):527–35. 10.1080/09638237.2019.1644490 31364902

[57] Fernández L , Fossa A , Dong Z , et al . Words matter: what do patients find Judgmental or offensive in outpatient notes? J Gen Intern Med 2021. 10.1007/s11606-020-06432-7. [Epub ahead of print: 02 Feb 2021]. PMC8390578 33528782

[58] Cromer R , Denneson LM , Pisciotta M , et al . Trust in mental health clinicians among patients who access clinical notes online. Psychiatr Serv 2017;68(5):520–3. 10.1176/appi.ps.201600168 28142383 PMC5411285

[59] DesRoches CM , Bell SK , Dong Z , et al . Patients managing medications and reading their visit notes: a survey of OpenNotes participants. Ann Intern Med 2019;171(1):69–71. 10.7326/M18-3197 31132794

[60] Blease C , Fernandez L , Bell SK , et al . Empowering patients and reducing inequities: is there potential in sharing clinical notes? BMJ Qual Saf 2020;29(10). 10.1136/bmjqs-2019-010490 32188711

[61] Gerard M , Chimowitz H , Fossa A , et al . The importance of visit notes on patient portals for engaging less educated or Nonwhite patients: survey study. J Med Internet Res 2018;20(5):e191. 10.2196/jmir.9196 29793900 PMC5992450

[62] Ancker JS , Barrón Y , Rockoff ML , et al . Use of an electronic patient portal among disadvantaged populations. J Gen Intern Med 2011;26(10):1117–23. 10.1007/s11606-011-1749-y 21647748 PMC3181304

[63] Klein JW , Peacock S , Tsui JI , et al . Perceptions of primary care notes by patients with mental health diagnoses. Ann Fam Med 2018;16(4):343–5. 10.1370/afm.2287 29987083 PMC6037508

[64] Kristiansen E , Johansen M , Zanaboni P . Healthcare personnels’ experience with patients’ online access to electronic health records: Differences between professions, regions, and somatic and psychiatric healthcare. SHI 2019. Proceedings of the 17th Scandinavian Conference on Health Informatics, November 12-13, 2019, Oslo, Norway. Linköping University Electronic Press, 2019:93–8.

[65] Strudwick G , Booth RG , McLean D , et al . Identifying indicators of meaningful patient portal use by psychiatric populations. Inform Health Soc Care 2020;45(4):1–13. 10.1080/17538157.2020.1776291 32603617

[66] Semahegn A , Torpey K , Manu A , et al . Psychotropic medication non-adherence and its associated factors among patients with major psychiatric disorders: a systematic review and meta-analysis. Syst Rev 2020;9(1):1–18. 10.1186/s13643-020-1274-3 31948489 PMC6966860

[67] Blease C , Dong Z , Torous J , et al . Association of patients reading clinical notes with perception of medication adherence among persons with serious mental illness. JAMA Netw Open 2021;4(3). 10.1001/jamanetworkopen.2021.2823 PMC7991965 33760088

[68] Blease CR , Walker J , Torous J , et al . Sharing clinical notes in psychotherapy: a new tool to strengthen patient autonomy. Front Psychiatry 2020;11. 10.3389/fpsyt.2020.527872 PMC7655789 33192647

[69] Salmi L , Dong ZJ , Yuh B , et al . Open notes in oncology: patient versus oncology clinician views. Cancer Cell 2020;38(6):767-768. 10.1016/j.ccell.2020.09.016 33035494

[70] Moll J , Cajander Åsa . Oncology health-care professionals’ perceived effects of patient accessible electronic health records 6 years after launch: A survey study at a major university hospital in Sweden. Health Informatics J 2020;26(2):1392–403. 10.1177/1460458219881007 31621459

[71] Hägglund M , Moll J , AAhlfeldt R-M . Timing It Right-Patients’ Online Access to Their Record Notes in Sweden, 2018: 336–40. 29677978

[72] Klein WMP , Ferrer RA , Kaufman AR . How (or Do) People “Think” About Cancer Risk, and Why That Matters. JAMA Oncol 2020;6(7). 10.1001/jamaoncol.2020.0170 32437492

[73] Shaverdian N , Chang EM , Chu F-I , et al . Impact of open access to physician notes on radiation oncology patients: results from an exploratory survey. Pract Radiat Oncol 2019;9(2):102–7. 10.1016/j.prro.2018.10.004 30342179

[74] OpenNotes . Implementing OpenNotes: Improving patient access to notes on patient portals - An OpenNotes White Paper, 2018. Available: http://www.opennotes.org/wp-content/uploads/2019/01/Implementing_OpenNotes_Improving_Patient_Access_to_Notes_on_Patient_Portals.pdf [Accessed 20 Jan 2021].

[75] deBronkart D , Sands DZ . Warner Slack: “Patients are the most underused resource”. BMJ 2018. 10.1136/bmj.k3194

[76] Blease C , Torous J . Opening mental health notes: 7 tips to prepare clinicians. psychology today, 2020. Available: https://www.psychologytoday.com/us/blog/digital-mental-health/202010/opening-mental-health-notes-7-tips-prepare-clinicians [Accessed 11 Dec 2020].

[77] Denneson LM , Pisciotta M , Hooker ER , et al . Impacts of a web-based educational program for veterans who read their mental health notes online. J Am Med Inform Assoc 2019;26(1):3–8. 10.1093/jamia/ocy134 30445648 PMC7647153

[78] OpenNotes . OurNotes for patients: creating notes with clinicians. opennotes.org, 2020. Available: https://www.opennotes.org/ournotes-patients/ [Accessed 18 Dec 2020].

[79] Kriegel G , Bell S , Delbanco T . Covid-19 as innovation accelerator: Cogenerating telemedicine visit notes with patients. NEJM Catalyst, 2020.

[80] Mafi JN , Gerard M , Chimowitz H , et al . Patients contributing to their doctors' notes: insights from expert interviews. Ann Intern Med 2018;168(4):302–5. 10.7326/M17-0583 29132154 PMC8650534

[81] Hoffman L , Wisniewski H , Hays R , et al . Digital opportunities for outcomes in recovery services (doors): a pragmatic hands-on group approach toward increasing digital health and smartphone competencies, autonomy, relatedness, and alliance for those with serious mental illness. J Psychiatr Pract 2020;26(2):80–8. 10.1097/PRA.0000000000000450 32134881 PMC7135933

[82] Casillas A , Perez-Aguilar G , Abhat A , et al . Su salud a la mano (your health at hand): patient perceptions about a bilingual patient portal in the Los Angeles safety net. J Am Med Inform Assoc 2019;26(12):1525–35. 10.1093/jamia/ocz115 31373362 PMC6857500

[83] Blease C , Salmi L , DesRoches CM . Open notes in cancer care: coming soon to patients. Lancet Oncol 2020;21(9):1136–8. 10.1016/S1470-2045(20)30423-X 32888448 PMC7462451

